# Remarks on the Gibraltar Epidemic

**Published:** 1831-07

**Authors:** D. Barry


THE LONDON
### Medical and Physical Journal.

389, VOL. LXVII.] JULY 1831. [61, *New Series.*

"For many fortunate discoveries in medicine, and for the detection of numerous errors, the world is indebted to the rapid circulation of Monthly Journals; and there never existed any work to which the Faculty, in Europe and America, were under deeper obligations than to the 'Medical and Physical Journal of London,' now forming a long but invaluable series."—RUSH.

### ORIGINAL PAPERS, AND CASES,

### OBTAINED FROM PUBLIC INSTITUTIONS AND OTHER AUTHENTIC SOURCES.

### GIBRALTAR EPIDEMIC.

*Remarks on the Gibraltar Epidemic.* By D. BARRY, M.D.

(Continued from page 494 of the last volume.)

WERE the germs of the late Gibraltar fever imported into that fortress in the summer or autumn of the year 1828, and afterwards propagated there?

Let us examine the sanitary history of the vessels which arrived at Gibraltar during the period specified, from countries where a disease identical with the late epidemic malady is indigenous, or, at least, of very frequent occurrence.

By the official returns of arrivals, deaths on board, and quarantines at Gibraltar, from 1st June to 1st September, 1828, called for by the commissioners of inquiry, in the spring of 1829, and laid before them by WM. SWEETLAND, esq. captain of the port, it appears that as many as ten deaths occurred during the summer, in vessels on their passage to Gibraltar, from yellow-fever countries, such as Cuba, and the ports of Spanish South America.

Natural deaths on board ships from suspected places are always, I believe, presumed, at the port of arrival, to have been caused by the disease against which precautions have been established: yet in no case were there any extraordinary measures of sanitary police adopted towards these vessels, except in that of the Swedish ship, the Dygden, and in her case only after her cargo had been landed. All the other ships, apparently under similar circumstances, were merely ordered a simple quarantine of twenty-one days, as will be seen by Dr. HENNEN's letter, to be hereafter quoted.

The Meta brig arrived on the 17th July, and the Hyperion

Rec'd: 81-36-5 (CONTIN.)
pp. 1-23, 96-107.

about the same time, from Cuba and the Havannah. They were both in Gibraltar with the Dygden, and were both suspected by the public. Mr. Cozens, the consignee of the Dygden, in a paper read by him, and afterwards given in to the board of commissioners, stated that the Hyperion was admitted to free communication, or pratique, on the fifty-fourth day after her departure from the Havannah. The Dygden, however, from the moment she entered the bay of Gibraltar, appears to have become an object of the strongest suspicion, and finally to have been pointed out, by public suffrage, as the "fons et origo mali." I shall therefore detail, in chronological order, the sanitary events connected with this latter ship, as far as I have been able to find them authenticated by official documents and oral testimony.

1st. The Dygden sailed from Havannah on the 12th of May, 1828, and arrived at Gibraltar on the 28th June of the same year, after having suffered the casualties enumerated in the following official paper, handed in by Mr. Lindblad, Swedish consul at Gibraltar, when giving evidence before the board of commissioners in 1829.

(COPY.)

"On the 12th August, 1828,* the master reported to me as follows, viz. That the following of the crew with which he left Europe had run away in Havannah: viz. one second mate, one cook, one cabin-boy, and eight seamen, (names given in the original.) That he had shipped in Havanah the following, viz. six seamen, (names given.)

"That, on the passage from Havannah, had died from sickness, Johan Sjôberg and Eric Johan Cajander, (seamen.)

"That he had discharged, and paid off here, the following, viz. five seamen, (names given.)

"That he had shipped here, for the run from hence to Cadiz, the following, viz. six seamen, (names given.)

(Signed) "JOHN LINDBLAD."

"*Gibraltar; 20th April*, 1829."

2. One of the sailors of the Dygden, named Roca, whose sister's case is alluded to in my last letter,† told his family, in Gibraltar, that he himself entered to serve in her, in the Havannah, directly from the hospital, and as yet barely convalescent from yellow fever, to fill up one of the vacancies occasioned by four or five of her crew having died of that disease, shortly before. This was given in evidence to the board of commissioners, by the uncle of Roca. Indeed, it is

* The very day the vessel sailed from Gibraltar for Cadiz.—D. B.

† See my official opinion in your number for November last, p. 395.

scarcely credible, that the cook, second mate, cabin-boy, and eight seamen, should have been all lost by desertion, in such a place.

3. Upon the arrival of the Dygden at Gibraltar, on the 28th June, Mr. Kent, the second officer of the quarantine department, went alongside, and ascertained, as stated by him to the commission of inquiry, that there were thirteen persons on board; that nine had been ill, and two died of fevers, on the passage; and that some of the survivors were looking delicate, or unwell, or words to that effect. The ship was conducted to the quarantine ground by Mr. Kent, was at first announced as consigned to Mr. Echicopar, but finally placed in the hands of Mr. Cozens. We hear nothing further of the Dygden until she commenced landing her cargo, on the twenty-first day of her quarantine, viz. 17th July.

4. The Dygden is again brought forward in the following official correspondence.

"To William Sweetland, Esq., Captain of the Port.

"*Gibraltar;* 31*st July,* 1828.

"Sir: I enclose a letter from Jasper Waring, esq., his Majesty's consul at Alicante, and beg you will be good enough to state all the particulars relative to the Swedish vessel now in this bay, from the Havannah, in order that I may communicate the same to that gentleman. (Signed)

"GEORGE DON,
"General and Lieut.-Governor."

"Certified to be a true copy,
(Signed) "J. Marshall,
"Military Secretary."

Enclosure above referred to.

"*British Consulate, Province of Valencia,*
"*Alicante; July* 22*d,* 1828.

"Sir: It having been reported, from what authority I do not know, to the Board of Health of this place, that a Swedish vessel had arrived at Gibraltar, from the Havannah, infected with the yellow fever, of which two of the crew had died,* the quarantine of eight days of observation, previously put on vessels arriving here from Gibraltar, has, consequently, been doubled. I, therefore, request that your Excellency will have the goodness to inform me, if any vessel has been at your port, or not, under the above cir-

* "A report of the kind did reach Gibraltar from the eastern coast, from Alicant, *not Valencia.*" (See Mr. Fraser's letter, in your March number, p. 213.)

cumstances, that I may be enabled to represent to the Board of Health here, the real state of the matter.

"I have the honour to be, &c.
(Signed) "JASPER WARING, Consul."

"To his Excellency, Sir GEORGE DON,
"Governor of Gibraltar, &c."

"A true copy, "D. BARRY."

### Reply from the Captain of the Port to the above.

"*Gibraltar; July* 31*st*, 1828.

"Sir: The inquiries of Mr. Consul Waring are, I presume, directed to the Swedish ship Dygden, Captain H. G. Yerle, master.

"This vessel arrived here from Cuba, on the 28th June, after a passage of forty-seven days, with a cargo of sugar and logwood: she lost two men on her passage, one on the 27th May, the other on the 1st June, both of fevers; but, since that day, the remainder of her crew have been perfectly healthy, and are so still. On her arrival, the ship was put under a quarantine of forty days, of which thirty-four have elapsed. The cargo being unsusceptible, is in course of landing. The vessel, under the inspection of two health guards, on board, has been once fumigated,* and will be admitted, please God, on the 6th August.

"I have the honour to be, &c.
(Signed) "WILLIAM SWEETLAND,
"Captain of the Port."

"To his Excellency Sir GEORGE DON,
"Lieutenant Governor, &c."

"A true copy, "D. BARRY."

On the 29th of July, Dr. HENNEN writes to Sir George Don, thus:

"I beg leave to state to your Excellency, that the reason why the Swedish ship Dygden, from the Havannah, was placed in quarantine for forty days, while all others from the same port were only ordered twenty-one, arose from her having lost some men on the passage."

5. Two health guards, it will be observed, are mentioned in Mr. Sweetland's letter of the 31st July, as having been placed on board the Dygden; but that gentleman omits to state, that these guards did not embark until the 27th July, nine days only before the vessel was admitted to free pra-

* The account given to the Board by the health guard examined upon this subject was, that several ounces of sulphur (I forget how many,) were burnt between decks, with wet straw; all the hatches being closely fastened down, and all the people below at the time, including "the man who lived to tell the tale."—D. B.

tique.* The motives of this extraordinary measure, thus tardily adopted, will perhaps be found in the following:

"To W. Sweetland, Esq., Captain of the Port, &c.

"*Gibraltar; 25th July,* 1828.

"Sir: The Spanish consul has just acquainted me, according to information he has received, it appears that a fever of a bad and complicated type had broke out in the island of Cuba, and rages at the Havannah. I communicate the above to you, for your information and guidance.†

(Signed) "GEORGE DON,
"General and Lieut.-Governor."

"Certified to be a true copy,
(Signed) "J. Marshall,
"Military Secretary."

Mr. Leach, clerk in the house of Mr. Cozens, to whom the Dygden, as already stated, was finally consigned, gave evidence before the Board of Commissioners, at their thirty-seventh sitting, that Captain Yerle, of the Dygden, reported his mate sick, by a note to the house about the 24th July, described the symptoms, and asked for advice and medicine. This note, which could not be produced to the Board, was shewn to Mr. Fraser, who sent salts and colocynth. Mr. Cozens himself, in a written statement given in by him, confirmed Mr. Leach's evidence, and specified the 24th July as the date of the report made by Captain Yerle, of his mate's illness.

6. Lieutenant and Adjutant, now Captain Harris, of the 43d light infantry, deposed to the Board of Inquiry, that he saw a mattress thrown overboard from the Dygden, whilst she lay in quarantine in the harbour of Gibraltar.

7. On the 6th of August, 1828, the Dygden was admitted to pratique, or free intercourse with the community, and on that day one of her people was brought to the wharf, with his arm in a sling, as a case of fracture, was admitted into the Civil Hospital, by Mr. Fraser, as "contusio brachii," and

* "Tenth. The period of quarantine shall be calculated from the day on which the health-guards are placed on board." (See Lord Chatham's proclamation, dated "Gibraltar, 8th October, 1824.")—D. B.

† The Dygden's bill of health will, no doubt, be here appealed to; but those who published that document ought to have known that foul bills are never given at the Havannah. When the existence of yellow fever can be no longer concealed, a note is added to the following effect, viz. "Some Europeans and North Americans are suffering from yellow fever." As well might we expect to see a foul bill of health from Cork or Dublin, because the typhus might happen to prevail in these cities, at the time the vessel, bearing the document, cleared out from their ports.—D. B.

appears on the books of that institution as discharged on the 10th of the same month.

8. The very next day, the 7th August, another sailor, named John Barrow, belonging to the Dygden, was brought sick to the same wharf: he complained of pains in his forehead and loins, both to Mr. Rombado, the inspector of strangers, and to Hospital-assistant Woods, who was sent to examine him, on the part of the health office. Mr. Rombado stated to the Board of Commissioners, that when this man was describing his complaints to him and Mr. Woods, he drew his hand across his brows, and round his loins, to indicate the situation and severity of the pains he felt. A scrap of paper, purporting "that this man laboured under no complaint likely to endanger the health of the garrison," was signed by Mr. Woods; and on that authority he was permitted to pass into and through the town, to the Civil Hospital, situated in the midst of it. It is clear, however, that a certificate, so loosely worded, and so totally deficient in specific information, could convey no adequate notion as to the consequences which might attend the admission of a case like the present into the bosom of a healthy population, circumstanced as that of Gibraltar was at the time, except to those who might happen to be acquainted with Mr. Wood's own notions and experience on the subject of medical police. The man was received into the Civil Hospital, where his name and disease stand registered in two different books, in the handwriting of Mr. Fraser, the surgeon of that establishment, as follows:

1st. In the daily admission and discharge book, thus: "John Barrow, 7th August; febris intermittens."

2dly. In the diary of in-patients, thus: "8th August, John Barrow, aged thirty-six; febris intermittens; admitted yesterday, from the Havannah, where he had fever. Spoon diet, soup." He is returned discharged on the 10th August.

9. The bag of foul clothes brought on shore from the Dygden, by the sailor Roca, and the consequences which followed the consignment of these clothes to his sister,* are

* See this Journal for November 1830, page 395. This girl, Maria Roca, was servant to Mr. Bellardo, and, according to her own and her master's testimony to the Board of Commissioners, received a bag of clothes to be washed from her brother Louis, who had arrived in the Dygden, and who, from having himself also lived as servant, when a boy, in Mr. Bellardo's family, was well known to every member of it. Maria shook out and counted the things; but Mrs. Bellardo having refused her permission to have them washed in her house, on account of their having come from the Dygden, the bag was hung up in an open terrace on the roof. Louis, after mentioned in my last, as well as the illness of Miss Teste, who washed the clothes of her brother the health-guard, brought on shore about the same time.*

10. Mr. Larios and others deposed to the Board, that the health-guard Teste, in their presence, cursed the Dygden, early in the epidemic, and repeatedly declared that she had brought the sickness to the garrison. It is important to remark here, that Mr. Teste, in his examination before the Board, gave the strongest proof that he was at least an unwilling evidence, and that he concealed as much of what he knew as he could with decency; no doubt, from a wish not to compromise the department to which he belonged.

11. One of the men shipped in Gibraltar on board the Dygden, for the run to Cadiz, stated to the Board, that another of their number was taken ill after he embarked, was confined to bed, and had medicine from the captain; and that clothes and other effects were thrown overboard whilst the ship lay in quarantine in Cadiz harbour.

The declaration contained in the subjoined note will further confirm the above evidence.† But to sum up.

having remained three or four days at Mr. Bellardo's, found a berth on board another ship, took away his bag of clothes, and quitted Gibraltar. Maria was taken ill on the 20th of August, and was three days in bed, attended by a Dr. Mortera, who at first thought her liver affected, but afterwards declared to several persons that her symptoms, in August, were the same as those which he saw others suffer in the course of his practice in the subsequent epidemic. He had never seen the disease before, and died of it himself not many weeks after. This case was not reported to Dr. Hennen; but the girl suffered another severe attack at a later period, which was also thought to be of the genuine type. She was the only individual of the Bellardo family who had not passed the disease in some former epidemic.

It is a curious coincidence, if it be nothing more, that the son of Mr. Baro, lottery-office keeper, residing on the floor under the Bellardo family, was the first individual attacked after Maria Roca in that part of the town. This child was much caressed both by Maria and her brother, during his visits to the house. He sickened on the 8th September, and died, as did Mr. Baro's second child, a few days after.—D. B.

* In reply to the circular of the Board, relatively to first cases, Dr. Diaz, who attended Miss Teste in her illness, writes thus: "The observation made by me on the patient Teste, her long convalescence, and all that has happened since in this garrison, leave no doubt with me, that the illness of this lady has been the Typhus icterodes; and I consider her as the first patient under my assistance, in the late past calamity. God preserve you many years.

(Signed) "JOHN DIAZ."

"*Gibraltar;* 30*th January,* 1829."

"To Dr. Barry, Secretary to the Board, &c."—D. B.

† Vincenzo Daveggia, an Austrian seaman, now belonging to the town vessel called the Diana, states that he was one of the six men engaged in Gibraltar to assist in navigating the Swedish vessel Dygden from Gibraltar to Cadiz, in the month of August last: that, on their first arrival there, the health officer refused

The Dygden leaves Europe with a northern crew, susceptible, as allowed by all, in the very highest degree, of yellow fever. In the Havannah she loses eleven of this crew, northern names to a man, except the cook and cabin-boy: the whole eleven by desertion, according to the captain; four or five by yellow fever, according to Roca. About the middle of May she sails for Europe, and at sea, from the 14th to the 20th day after her departure from Havannah, she loses two out of nine cases of fever; northern names also. Arrived at Gibraltar, she is first announced as consigned to one house, then handed over to another, and after twenty-one days' observation, begins to land her cargo. Seven days after this operation had commenced, viz. on the 24th July, the captain, whose interest it most assuredly was to conceal any illness, unless it were of a serious nature, that might occur on board his ship during her quarantine, reports his mate sick to his consignee, who refers to receive them, stating that they must perform quarantine at Mahon; but, after many examinations and applications on the part of the captain, they were allowed to perform fourteen days' quarantine at Cadiz: that, towards the close of the quarantine, one of the six men hired at Gibraltar was taken ill; the captain declared that his bowels were only out of order, but the patient himself told his companions that it was not true, for he had a pain in the head and stomach, *(mal di pancia:)* he was in bed two days, attended by the captain, who gave him various medicines; got up on the third, but appeared weak and very pale, and unable to go out for two days afterwards. When this man was taken ill, the captain seemed alarmed and agitated, and made them strike all the masts and yards, for fear of being sent to Mahon. On the day on which they got pratique, when the crew were called to the side of the ship, for the health officer's inspection, the captain made the sick man in question face towards the bow of the ship, for the purpose of concealing his countenance.

Whilst the Dygden was in quarantine at Cadiz, a dispute having arisen between the six men from Gibraltar and the crew, the captain, coming amongst them, addressed the Gibraltar people, and said, "As for you, I have left a present at Gibraltar, which will never be forgotten." About a week ago, deponent suddenly met the captain of the said vessel in a street at Cadiz, when, in the course of conversation about the epidemic fever of last year, deponent remarked what he (the captain) had said about the present he had left in Gibraltar, on the night of the dispute on board: to which the captain immediately replied, "Yes, I did say these words to you; and you now see that I did actually leave a present at Gibraltar, which will not soon be forgotten."

To the truth of the whole of the above particulars, Vincenzo Daveggia and his companion, (another of the six men hired at Gibraltar,) Juan Arduino, who accompanied him to the office, both declared that they were ready to swear.

(Signed) "VINCENZO DAVEGGIA,
"GIO. BATTA ARDUINO."

"Taken before Colonel Falla, town major, and Alexander Broadfoot, esq. principal medical officer at Gibraltar, this 27th day of June, 1829.

(Signed) "D. FALLA, Town Major,
"ALEX. BROADFOOT, Inspector of Health and P.M.O."

the case to his own doctor, Mr. Fraser. Observe that had this report been communicated to the Health-office, the ship's quarantine must have recommenced, to be counted from the date of the mate's attack, and so of every successive case. The Spanish consul at Gibraltar being, from the information he possessed, justly apprehensive of danger, writes to Sir George Don on the day after the mate's illness. The Board of Health of the province of Valencia officially announce to the British consul at Alicante, that a Swedish ship had just brought the yellow fever to Gibraltar, and immediately double their own measures of precaution. Sir George Don announces these facts officially to the heads of the health department of his government, and these gentlemen declare the suspected vessel to be perfectly free from the seeds of the disease apprehended, because none of her people had been officially reported sick to them during her quarantine. To quiet public feeling, however, health guards are placed on board at the eleventh hour, and nine days after, viz. on the 6th August, 1828, the Dygden is admitted to free intercourse with the community of Gibraltar, in the face of the letters just quoted.

On that very 6th of August, and the next day, two of the Dygden's crew are sent sick to the Civil Hospital: one pretending to have a broken arm, the other acknowledging febrile symptoms, and registered in Mr. Fraser's own hand, on two successive days, as "febris intermittens from the Havanah, where he had fever." Now, to justify Mr. Fraser's diagnosis, which, from the entry on the second day, he seems to have considered certain, John Barrow should have enacted "the drama of fever in the interim, the three acts, cold, hot, sweating:" but as the drama was not repeated, and the Dygden being rather in a hurry to get off, (she sailed for Cadiz on the 12th,) John Barrow and his companion were struck off the books on the 10th of August.

We have now authentic proofs, that of the fifteen individuals who sailed from the Havannah in the Dygden, on the 12th of May, nine were attacked, and "two died of fevers" on the passage, and that two at least were ill between the time of her arrival in Gibraltar, and her departure for Cadiz on the 12th August.

Such is the chain of evidence, and I defy the abettors of local origin to unrivet a single link of it, upon which I founded the explanatory remarks attached to my official opinion, given as a member of the Board of Commissioners in 1829;* and

* See your November number, p. 395.

such are the proofs, upon which I now repeat the assertion, that the Dygden affords all the characters of a pestilential ship, from the moment she is first heard of at the Havannah, during her passage, all through her stay at Gibraltar, and until she is lost sight of at Cadiz. Let her history be compared with the importation of the yellow fever by the Bann sloop of war into the island of Ascension in 1823, so candidly recorded by Dr. (now Sir WILLIAM) BURNETT, and so ably commented on by Sir GILBERT BLANE. Let it be compared with that of the Donostiarra, the ship which both contagionists and their adversaries unanimously agree introduced the yellow fever into the town of Passages, in the north of Spain, in August of the same year.*

In vain will the special pleaders urge, that the Donostiarra had no sick on board, and therefore could not have spread the disease by contagion; that she was a floating marsh, a magazine of malaria. Be it so: but then, these casuists must allow, that the sailor Roca and his bag of foul clothes, John Barrow, his companions, and their baggage from the Dygden, were also little moving stores of portable malaria. "Vehiculum duplex est, aër et fomes aliquis."

There are some very curious points of resemblance between the sanitary histories of these two ships. The Donostiarra sailed from the Havannah in the beginning of June, with a crew of *fifteen persons*, five passengers, and a cargo of sugar, coffee, &c.; after having been ten days at sea she lost a man; and, after a passage of thirty-five days, arrived at Corunna, from whence, after having passed a quarantine of ten days, she sailed for St. Ander, where she anchored, staid a few days, and finally arrived at Passages on the 2d of August, all well, and, like the Dygden, with a clean bill of health. Under these circumstances no quarantine was imposed; vessels from St. Ander being always admitted into Passages without any questions. The landing of the cargo immediately commenced, and was completed by the 16th, without any of the people employed having been taken ill; but, on the 17th, the customhouse guard, Ali, who had passed several days on board, died with black vomit, and, on the 20th, the shipwright, who had surveyed the vessel; next the carpenters employed in repairing her, two boat-women, (sisters,) who conveyed persons to and from the ship; the chocolate seller, who had gone on board, all died within a few days; and under symptoms exactly similar to those of Ali. Some of the remaining carpenters refused to continue the repairs, retired, and escaped; whilst fresh

* This little town is situated in 43° 21′ north latitude.—D. B.

hands, induced to come on board by large offers of increased wages, shared the fate of their predecessors.

On the 10th of September, the town-council, the faculty, and the public, unanimously decided (and indeed how could they have decided otherwise?) that the disease was the yellow fever, and that its source was the ship Donostiarra. She was accordingly ordered to be sunk. Still the consignee of the vessel, Mr. Quéheile, his friends, and his doctor, Zubeldia, stoutly maintained that the disease was of local origin; that the Donostiarra was innocent, because the persons who had assisted to land her cargo were not the first affected, because she brought a clean bill of health, because she had had no sickness since the death of the man at sea, about the middle of June, and, finally, because the captain solemnly declared that that man died from indigestion, after having eaten too largely of pine-apple and rum.*

Personal interests, or bad passions, may distort these facts; theory may dress them up to suit her own hypotheses; but the following simple truths must remain unimpeached, and unimpeachable, for ever.

1st. That the Donostiarra brought the germs of the yellow fever, *with a clean bill of health,* across the Atlantic.

2d. That these germs were propagated amongst a population previously healthy, and in a place where that disease had never been known before.†

3d. That, notwithstanding the submersion of the ship (equivalent to the departure of the Dygden,) and the flight of a large portion of the inhabitants of that small town, 101 individuals were attacked, and forty died, between the unloading of the infected vessel on the 16th of August, and the 20th of October following.‡

In that very town of Passages, in the summer and autumn of 1813, when Gibraltar, by the way, was suffering under her third epidemic, I had charge of the whole of the sick and

* It will be seen further on, that the first cause assigned for the death of the Fani boy and girl, was their having eaten too freely of green figs.—D. B.

† The following extract from the celebrated Huxham will prove that pestilential fever may be imported by sickly ships, and propagated even in this country, amongst a population previously healthy. "Ineunte vere ad hunc portum (Plymouth) appulsæ sunt naves duæ bellicæ (Panther et Canterbury quarum posterior mox a Mare Mediterraneo.) Ex his Ægroti plusquam ducenti ad terram protinus delati sunt, horumque pars major febre maligna, imo pestilente laboravit; hinc in vulgum quoque sparsa maximam edidit stragem." (Huxham, "Prolegomena Plymouthi facta, ann. 1728 ad 1737," vol. ii. p. 44.)—D. B.

‡ See Arruti, "Fiebre Epidemica de Passages," 1824; Audouard, "Recueil de Memoires," 1825.

wounded of the Portuguese army. A great number of the sick of the British army was also there, and though these, with the followers and shipping of both armies, increased the population, perhaps, twenty-fold, yet there was no yellow fever. The infected bale, the "fomes aliquis" was alone wanting to complete that combination of causes, the inevitable effect of which would have been an epidemic of the disease in question.

Regretting that the limits of a journal will not admit of my pursuing this branch of the subject further, for the present, I shall now address myself to the second leading question, viz. the disputed contagiousness, or communicability, of the late Gibraltar fever. Here I shall strictly confine myself to local facts and documents, considering them of more importance towards settling this question, than citations from the events and records of other epidemics, however high the authority, or however striking the analogy upon which these citations may be offered.

### *Contagion and Non-contagion of the Gibraltar Fever of* 1828.

The only arguments adduced in the course of this controversy against the doctrine of contagion, as applicable to this disease, are contained in the following assertions, which have been published to the world as so many facts.* These assertions I shall now contrast, individually, with authentic records of the events to which they refer; records drawn up long before this controversy was thought of, but which must be utterly unknown to, forgotten, or overlooked by Mr. Fraser and his abettors, or supposed perhaps, by these gentlemen, to be unknown to me.

1*st Assertion.* "That, for the period of about one month from the commencement of the epidemic,† no hospital servant was taken ill *at all,* though in the closest attendance upon the sick."‡

* See Dr. Johnson's Journal for September 1830, p. 348.

† "But, to be more rigid regarding the military, let us take it (the commencement of the epidemic) from the 2d September, the day on which the first soldier attacked was received within the walls of the hospital." (See Mr. Fraser's Reply, April number, p. 314.

‡ See Dr. Johnson's Journal, as before.—D. B.

*Contrast No.* 1. *General Abstract of Military Hospital-Attendants employed in the Regimental Hospitals in Gibraltar, from 1st September to 10th December,* 1828; *shewing the number of these attendants attacked in each month of the epidemic. Taken from the original, official, nominal Returns, signed, as below, by the respective Surgeons in charge.*

| Corps and Regiments. | Number of Military Attendants. | Number attacked in each Month of the Epidemic. Sep. Attacks | Sep. Deaths | Oct. Attacks | Oct. Deaths | Nov. Attacks | Nov. Deaths | Dec. Attacks | Dec. Deaths | Grand Totals. Attacks | Grand Totals. Deaths | Signed by |
|---|---|---|---|---|---|---|---|---|---|---|---|---|
| Royal Ordnance | 22* | 8 | 0 | 7 | 7 | 2 | 0 | 0 | 0 | 17 | 7 | J. Halahan, Surgeon. |
| 12th Foot ...... | 23 | 2 | 1 | 16 | 7 | 5 | 1 | 0 | 0 | 23 | 9 | R. Amiel, Surgeon. |
| 23d R. W. F. ... | 16 | 1 | 0 | 11 | 4 | 2 | 2 | 0 | 0 | 14 | 6 | T. Smith, Surgeon. |
| 42d Roy. Highl. | 21 | 3 | 0 | 14 | 4 | 1 | 1 | 0 | 0 | 18 | 5 | J. M'Grigor, Ass. do. |
| 43d Light Inf.... | 27 | †1 | 0 | †19 | 9 | 0 | 0 | 0 | 0 | 20 | 9 | J. Gillkrest, Surg. |
| 73d .............. | 15 | 0 | 0 | 8 | 4 | 2 | 0 | 0 | 0 | 10 | 4 | G. Martin, Surgeon. |
| 94th .............. | 31‡ | 7 | 0 | 15 | 3 | 6 | 0 | 0 | 0 | §28 | 3 | E. J. Bulteel, As. do. |
| Totals ...... | 155 | 22 | 1 | 90 | 38 | 18 | 4 | 0 | 0 | 130 | 43 | |

* One woman, a nurse, included; had passed the fever in 1814, not attacked in 1828.

† Three men passed the fever in September, and one in October, before being employed: not included.

‡ Five attacked before being employed.

§ Nine relapses, nineteen primary attacks whilst attending the sick in hospital.—D. B.

N.B. The only relapses noted in the above returns are those of the 94th, with the exception of one in the 12th foot. In the 94th return, there are forty names, [see your February number, p. 7,] and yet there were but thirty-one individuals employed: the same person being two or three times entered, agreeably to the number of relapses for which he is returned.—D. B.

*2d Assertion.* "That, when the disease began to appear amongst them (the hospital servants), it was not until it had begun amongst the inhabitants of the south district, in which the hospital of the military is situated."*

*Contrast No.* 2. The civil population of the south district, where all the military hospitals were situated, amounted to 2,343. Their intercourse with the town, the supposed source of malaria, was unrestricted, but they never entered the hospitals. Of the two thousand thus circumstanced, fourteen died, or one in 143, between the 1st September and 15th October. The number *attacked* is unfortunately not given in the return signed by the town major, herewith transmitted, (marked No. 2.)

* Vide op. cit. ibid.

Of the attendants on the military sick during that time, the number of which could not have exceeded one hundred, cut off from all access to the town, but confined to their respective hospitals, eighteen, or nearly one in five, died, within the period mentioned.*

Of sixty-one civilians employed by the local government, to attend the military sick, from the early part of October to various periods in November, two only are stated, in the nominal returns, not to have passed the fever at some former period, and to have been attacked during their attendance; viz. one, in the 73d return, "was sick in town in October;" and one in the 94th return, with the following note opposite his name: "These men (viz. seven civil orderlies,) had passed the epidemic in 1813 and 1814, with the exception of Jose Lewis, who had an attack during the present malady.†

*3d Assertion.* "That the first hospital servants taken ill were the cooks and watermen, whose services did not bring them into the wards of the sick."

*Contrast* No. 3. In the regimental hospitals of Gibraltar, so long as their respective sick did not amount to twenty or upwards, the number of attendants allowed to each was five, viz. one sergeant or steward, one cook, one waterman, and two orderlies or personal attendants.‡ Of the twenty-two attendants attacked in September, the first month of the epidemic, fifteen are returned as personal orderlies, four as cooks, two as watermen, and one as hospital sergeant. The first hospital servant, a cook of the ordnance, was attacked on the 5th September: eight cases had been previously admitted into that hospital; the first on the 1st September. The second hospital servant attacked was an orderly of the 94th regiment, on the 6th September, the second case in that corps.§

The military attendants on the sick, who had no attack during the epidemic, viewed as cooks, watermen, and orderlies, stand thus, according to the original nominal returns: viz. of the five who escaped in the ordnance hospital, one was a cook. In the return of the 23d regiment, the employments of the attacked servants are not specified. Of three escapes in the 42d hospital, one was a waterman. Of five escapes in the 73d, one was a cook and one waterman. Of three escapes in the 94th, one was cook, one waterman.||

* See the returns of military and civil hospital servants, marked No. 1.—The documents referred to are in our possession.—Editor.

† See the 94th return.

‡ One orderly to every ten sick.

§ See the general abstract, No. 1, and nominal returns, ditto.

|| See the returns of servants.

*4th Assertion.* "That according to a nominal list of sixty-nine men of the 43d regiment, who, during the *first month* of the epidemic, were exposed in the wards day and night, in parties of from four to six each, for the space of twenty-four hours, the number of them attacked was found, at the close of the epidemic, not to have exceeded the general mass of the regiment attacked."

*Contrast No.* 4. The above statement, purporting to be derived from the medical history of the gallant 43d, as well as the preceding arguments and assertions, seems to be grounded on such vague, unauthenticated information, that I shall notice it but slightly at present, for the reasons already assigned.

Mr. Fraser was not in any manner attached to the 43d, and cannot be justified in making any positive statement regarding the health of the regiment, unsupported by official documents.

The nominal return of admissions into that hospital, from 1st September to 20th December, signed by the surgeon, shews that the second epidemic case occurred on the 12th September, and that the whole admitted up to the morning of the 22d amounted to twenty-six, requiring, at most, three effective orderlies: therefore "the experiment," as it is called at page 316 of your April number, could not have been even begun, under any justifiable pretext, much before the end of the first month (September), nor completed on sixty-nine persons, at the rate of five per diem, much before the middle of the second month of the epidemic (October,) when the whole rock was but one hospital.

*5th Assertion.* "That it appears, that in hospitals placed out of the influence of the infected atmosphere of the rock, no servant was attacked, though several were exposed who were susceptible in the sense of not having had the disease before. This was shewn to be the case in the hospital establishments of the 73d and 94th, removed about the middle of the epidemic season, to Windmill-hill barracks.*

*Contrast No.* 5. On the 7th October, 1828, Windmill-hill barracks were ordered to be prepared for the reception of the sick. On the 14th, Dr. Hennen writes thus to the military secretary.

* The above five assertions, copied *literatim* from Mr. Fraser's first controversial paper, in Dr. Johnson's Journal, are all again reiterated by him, if possible, in a still more unqualified manner, at page 317 of your April number, where he concludes thus: "Let these facts, which, I venture to say, will bear *any examination*, be compared with the manner in which Dr Barry has dressed up the statement of the military hospital attendants, and it will be seen at once whether he has, or has not, been misleading both the profession and the public." I freely subscribe to this proposition.—D. B.

"The Naval Hospital having been evacuated by the 73d and 94th regiments, I shall be enabled to give over considerable accommodation upon the upper floor to the other regiments; but as it is a matter of *vital importance* that crowding should be avoided as much as possible, within the precincts of the building, I beg to submit, for his Excellency's consideration, that no slight cases, such as ulcers, ophthalmia, &c. be sent into the Naval Hospital.*

(Signed) "J. HENNEN."

"To Lieut.-Colonel MARSHALL."

You will perceive, by the nominal returns herewith sent, from which the general abstract No. 1 was made out, that, of the military attendants of the 73d regimental hospital, on Windmill-hill, six were attacked, and two died, from 16th October to 10th November, the dates of the first and last seizures at that station.

That, in the 94th hospital, at the same station, six attendants were attacked, and one died, from the 14th October to the 17th November, not including five relapses returned in this hospital within the period; making, in one month, twelve primary attacks, and three deaths, in Windmill-hill, amongst the hospital attendants of two battalions.

That, of the six attacked in the 73d hospital, two were cooks, and four personal attendants. Of the six in the 94th, one served as cook, two as watermen, and three as personal attendants on the sick.

If events such as are asserted in the above extracts would, and I grant that, fully authenticated, they would furnish strong statistic proofs that the disease to which they refer could not have been contagious, it follows, that their complete refutation, and the facts of a converse nature, just established upon the most irresistible evidence, are equally strong proofs that the Gibraltar fever of 1828 was contagious in that place at least, whatever properties it may have manifested elsewhere.

In the discussion of questions like the present, statistic facts are of such paramount value, that I shall venture to state one or two more. "A single row of figures clears away a whole forest of prejudices," as observed by the able reviewer of Dr. HENNEN's posthumous works, in your Journal for December last.

* Symptoms of a persuasion "of the possibility of the disease becoming contagious, from numbers being crowded together," "but that, after sufficient observation, he (Dr. H.) maintained an opinion of this kind, does not at all appear to have been the case." (Mr. Fraser's Reply, in your March number, p. 206.) Dr. H. survived the writing of the above letter but twenty-one days. The opinions of this distinguished surgeon on the subject of contagion shall be noticed hereafter, when I trust their uniformity, from the year 1826, will be demonstrated.—D. B.

The strength of the garrison, on the 1st September, 1828, the day on which the first soldier was taken ill of the disease, was 3781.*

Of these, 1436 individuals are stated to have been attacked before the 21st of December following, as appears from the original, nominal, official returns, signed by the surgeons of corps, giving the names of all men attacked with the fever in their respective regiments, during that period. One duplicate name is deducted from the return of the Royal Ordnance; sixteen from the return of the 23d; seven from that of the 43d; and thirty-three from that of the 94th; all entered a second or a third time, as relapses.

Three thousand seven hundred and eighty-one natives of cool latitudes, then, were shut up in Gibraltar during an epidemic, in all the health and freshness of manhood, to be used, as it were, for the purpose of testing, by positive experiment, the doctrines of contagion and local origin, as applied to the fever which had so often ravaged that insulated spot. The steps and the results of that experiment were the following.

1st. One hundred and fifty-five individuals, including cooks, watermen, &c. were employed, as stated in the abstract just given, for various lengths of time, and at various periods of the epidemic, to attend upon the sick; they were, in fact, exposed to the direct influence of contagious effluvia, if such existed, but were withdrawn, for the time, from night-duties out of doors,† from insolation, fatigues, the drains in town, and other supposed local causes. One hundred and thirty of the individuals thus circumstanced were attacked: in other words, less than one in six of them escaped infection.

2d. The remaining three thousand six hundred and twenty-six were exposed for the whole of about every third twenty-four hours, on duty, in the town and territory, not only to all the local causes from which the attendants on the sick were, for the time, withdrawn, but also to every species of malaria and atmospheric vitiation imagined by the abettors of local origin. They were, however, less frequently, and less directly, subjected to the influence of contagious emanation, which was brought into contact with their lungs in a less concentrated shape, suspended in the warm, unagitated atmosphere around the crowded hospital, and the private dwelling of the sick.

* See the return herewith sent, (Copy, No. 3.)—D. B.

† "I should say, as far as my own observation goes, that four fifths were attacked at night; and, as I have already observed, some particular guards sent more men to hospital than others." (See Edinburgh Med. and Surg. Journal for January 1831, p. 40; "Dr. Smith on the Gibraltar Fever of 1828.")—D. B.

One thousand three hundred and six of the individuals thus circumstanced caught the disease: in other words, nearly four in six of them escaped infection.

But, to avoid all sources of fallacy and cavilling likely to arise from the various nosological views adopted by the different regimental surgeons; from the mystifying distinctions of single and double relapses; from the same individual being twice or thrice named in the same return; let us take our estimate from the simple element of the number of rank and file who perished during the epidemic. In this there can be no error.

| | | |
|---|---|---|
| Strength of the garrison, on 1st September, 1828 . . . | | 3781 |
| Of 155 hospital attendants, there died, up to 24th Dec. or 1 in 3. | 43 | |
| Of 3626 not hospital attendants, there died, up to ditto, or 1 in 9, rejecting fractions. | 393 | |
| 3781 Total died . . . | 436* | |

From the above experiments, then, it would seem that the effects of the poison exhaled by the sick of the Gibraltar fever, upon the susceptible healthy subject, bore, like the effects of most other poisons, a direct proportion to the concentration of the dose, and the length of time during which it was applied.

*Contagion of the Gibraltar Epidemic of* 1828, *illustrated by the Sanitary History of Families and Individuals.*

I shall now pass from general to particular facts, with a view to ascertain whether such connexion can be traced between individual attacks of the fever, as would justify the conclusion, that it was propagated from person to person, from family to family, after the manner of contagious diseases. The earliest period, that from the admission of the ship Dygden on the 6th August, to the placing of the cordon by the Spaniards on the morning of the 5th September, is, for many reasons, the most favorable for this kind of investigation. The usual relations of society had not yet been disturbed; the sick and the healthy had not been mingled together by the alarming and tumultuous turning out to camp on the first day of the cordon: besides, up to that moment, the disease, which had been spreading exclusively amongst the civil population, was looked upon, by the medical authority, as nothing more than the ordinary "bilious, autumnal remit-

* See the General Return.

tent fever" of the country: there had been, as yet, no difference of theories, or opinions, amongst the faculty; the records, therefore, of these times are the most unprejudiced, the most unobjectionable, and certainly the most valuable, evidence we now possess.

Of this description are the following "Morning States" of the progress of the fever, made out from actual observation each day, during the first week of September, under the immediate personal inspection of Dr. Hennen. They formed, at the time, the basis of all his official statements on this subject, and derive no small additional value from the well-known accuracy and unimpeachable integrity of Mr. Tucker, who drew them up, and now certifies their authenticity.

RETURN NO. 2.

"*Morning States of Fever Cases (Bilious Remittent); Gibraltar,* 1*st September, nine o'clock, to* 7*th September*, 1828, *inclusive.*

September 1st, 1828. Died yesterday and this morning, three; remaining, ten.

One from Haines' Shed, road to Flat Bastion. One woman from Victualling-office lane. In District No. 24, one Christian and two Jewesses. One Christian convalescent, widow of Mr. Duguid's servant, John Whitelock. Four children, living next room to above. Total, ten.

Died yesterday in hospital, Mrs. Silcock, admitted from shed next to above servant's residence, on 29th August. John Whitelock died in District No. 24 this morning.

*Recapitulation of deaths:* Servant removed from Mr. Martin's house, one; above, two; total, three. (Signed) R. TUCKER, P. CLERK.

True copy, R. Tucker, Acting Deputy Purveyor.

2d. Admitted, four; remaining, fourteen.

One boy of sixteen, and one girl of thirteen or fourteen; a servant to Hassan; a servant to Mr. Fraser, who has been ill more than a week, treated in his own house.

*Mem.* The man in hospital is now moribund. The woman, Protestant nurse, who attended on Mrs. Silcock, who died in hospital, is in a very doubtful state; irritable stomach. (Signed) R. TUCKER.

True copy, R. Tucker, Acting Deputy Purveyor.

3d. Admitted, six; died, two; remaining, eighteen.

Died since last report, Gulierme Loary, in hospital; admitted from District No. 24, Haines' Shed. A Jewess girl, who has been attended at her own house in above district, by a private practitioner.

Names of remaining in hospital: P. Androbet, Catholic; M. Bocasis, Hebrew; S. Benhaim, ditto; John Loary; Mrs. Flinn; Sheriff, Peré, Ristano, and Benhim, children. In districts: Hassan's servant; Mrs. Smith, Mrs. Leahy; Mr. Fraser's servant woman; a Jew girl; Mrs. Leahy's four children. (Signed) R. TUCKER.

A true copy, R. Tucker.

**Sept. 4th. Admitted, three; discharged, three; remaining, eighteen.**

Admitted (hospital): Triani, Catholic, from Devil's Gap; Benures, Jew, District 24; District-sergeant King.

Discharged: Hassan's servant; Mr. Fraser's servant, well; a Jew woman, not being attended by medical officer.

*Mem.* In future, none will be included as sick in districts that are attended by private practitioners; of which, it is understood, there are several.

A true copy, R. Tucker. (Signed) R. Tucker.

**5th. Admitted, three; discharged, two; died, two; remaining, seventeen.**

Admitted into hospital: Godsell, a Protestant, from district No. 24; Safredi Catholic, fever supervening on hepatitis; Mezod Benhaim, Jew boy, from the infected house, son of the old Jew already in hospital, making three of the same family

Discharged: Sherriff, a native girl; Benures, a Jew.

Died: Bocasis, a Jew, from the infected house; Godsell, a Protestant, from District No. 24, who died in about fifteen hours in hospital, with all the marks of the disease, black vomit, fatuitous appearance of countenance, &c.

A true copy, R. Tucker. (Signed) R. Tucker.

**6th. Admitted, seven; died, one; remaining, sixteen.***

Admissions: From south, from a garden, one; District 24, six; total, seven.

Died: Guliermo Loary, son of John or Juan Loary, the man admitted from the iron-roofed shed in District 24, and who died 2d September.

*Mem.* Mr. Francis' daughter died last night in No. 24.

A true copy, R. Tucker. (Signed) Richard Tucker.

**7th. Admitted, twelve; died, two; remaining, twenty-six.**

Admitted from various districts.

Deaths: Francisco Triani, from District No. 24; James Keys, employed in the commissariat. (Signed)

J. Hennen, Inspector of Hospitals.

One additional, reported by Mr. Mathias.

A true copy, R. Tucker, Acting Deputy Purveyor.

Amongst the early cases, not noticed in the above Morning States, were two young persons, named Fennic, or Fani, born on the Rock, who died on the 17th and 20th of August. They were the son and daughter of a smuggler, and their elder brother kept a bumboat. It has been most erroneously stated by Mr. Fraser, that Mary Parody gave evidence as to the nature of their disease: it was the woman Villalonga,† who lived on the same floor, and next room but one to them, who deposed, that she saw the girl vomit black; and both she and the woman Salinas, that the girl, then ten years of age, told each of them separately, that she and her brother had

* Out-patients omitted.—D. B.

† This woman saw the Fani children frequently during their last illness.

been with their father on board ship the Sunday before her brother was taken ill, that they had eaten and drank there, and that her father had sold some segars on board. This statement was most fully confirmed to the Board by the boy Cafiero, who was one of the party on board ship, and was taken ill of the same disease at the same time with the Fani children.* This boy further stated that old Fani, nicknamed Malta, gave him a piece of money (a *pecetta*) not to tell where they had been. Gregorio Masa, master of a vessel, stated to the Board, that the bodies of the Fani children were yellow when he placed them in their coffins. A further evidence, if any were necessary as to the nature of their disease, is adduced by Mr. Fraser himself, with his usual naïveté, from the unwilling testimony of their mother, and the declaration of their medical attendant; viz. that they died of a *tabardillo*, which means a malignant fever, attended with red blotches resembling the bites of the *tabanus*, or ox-fly.†

It is not a little remarkable, with regard to the Fani children, that, though their rapid decease excited the attention of the chief police magistrate, Colonel Falla, sufficiently to induce him to have their parents and their physician, Dr. Lopez, examined on the subject at his office, when he was informed that their death was caused by eating green figs; yet that Dr. Hennen, chief of the health department, nowhere notices either their illness or deaths, though he includes, in his reports, cases simultaneous with, and deaths from presumed apoplexy, falls, and childbirth, about the same time. The same remark may be made with regard to the illness of the health-guard of the Dygden's sister, who was attacked on the 21st August, but who is not mentioned, as far as I am aware, by Dr. Hennen. Yet both Doctors Dias and Mery state, in their replies to the circular of the Board as to first cases, that they reported her's as a suspicious case, on the 25th of August.

16th August, 1828. The words "Mr. or Mrs. Whitelock, Febris," appear, on this and the following day, at the head of two prescriptions in the Dispensary out-patient book of the Civil Hospital. Whether these prescriptions refer to the wife, or the son of John Whitelock, Mr. Duguid's coachman, to be mentioned hereafter, I have not been able to learn. Most probably to the former, from the quantity of castor oil ordered (℥ss.) Certain it is, however, that neither Mr. Fraser nor Mr. Wilson, the surgeons of the hospital, make any allusion to this case of fever, either in their replies to the circular

* See Cafiero's case in your February number, p. 2.
† See the Dictionary of the Academy.—D. B.

of the Board, or in what they have since published on the epidemic.

Mrs. Whitelock states that she did not herself go to the hospital, though the surgery man says he recollects to have seen her there, but that her late husband was in the habit of taking one of the children there occasionally, for weeks before he was attacked himself; the boy's health being delicate at the time. That latterly the child became feverish, and turned yellow; but that he was nearly well when his father was seized with his fatal illness, on Wednesday, the 27th August. According to Mrs. Whitelock's own account, she was attacked the next day, the 28th; but, in the official "morning state of fever cases, dated nine o'clock, 1st September," she is returned "convalescent."*

There is a note appended to a prescription written for Whitelock the father, in his own apartment, on the 1st September, in Mr. Wilson's handwriting, thus: "Died while prescribing." The house in which this poor man resided up to his death, has been ever since pointed out, and was even then considered, as a focus of infection, or contagion: it is modern, well constructed, spacious, and airy.

As the following letters place the circumstances of this and another important case beyond the possiblity of cavil, and are likely to be often quoted, I shall here transcribe them.

"*Circular; Spanish Pavilion,* 28*th January,* 1829.

"Sir: I am directed by Dr. Pym, president of the Board of Commissioners, now sitting at the Court-house, for the purpose of inquiring into "the origin of the disorder which lately prevailed here," to request that you will have the goodness to transmit to me, by Friday next, a return of the first case, with name, date, and residence, which you may have treated, or which may have come to your knowledge, in this garrison, during the last year; and

* Gibraltar, —— 1829. Mr. Leahy, now servant to the American consul in Gibraltar, stated to the Anglo-French commission, that, about the end of July, 1828, he was intimate with a person named Kelly, then steward of the brig Meta, last from Cuba, who had formerly been the fellow-servant of Whitelock, in Mr. Duguid's family, of this place; that, in the beginning of August, he went on board to see Kelly, who gave some linen to Mrs. Whitelock, to be washed; that Kelly looked yellow and sickly, and said he had been very ill on his passage here; that Mrs. Whitelock had been brought to bed, about three weeks before she had Kelly's linen to wash; that some of the Jews up stairs, in the house where he and Whitelock lived, had been ill about the 17th August; that they themselves told him so, as did also their washerwoman, Josepha. Mrs. Whitelock acknowledges that her husband had been invited to go on board to see Kelly, but thinks that he did not go. Kelly sailed from hence in the Meta, on the 10th August.—D. B.

which you may have considered identical with the fever which so lately prevailed here in an epidemic form.

(Signed) "D. BARRY, M.D.
"Secretary to the Board."

"To PETER WILSON, Esq. Surgeon, &c."

Mr. WILSON's reply to the above:

"*Civil Hospital;* 31*st January*, 1829.

"Sir: In reply to your letter of the 28th instant, I have to acquaint you, for the information of the Board of Commissioners, that the *first case I saw* of the disease in question was Mrs. Martin's servant maid:* this was on the 27th August, and she *lived*† in a low room in district 26, not far from Mrs. Martin's house. The case I first treated was William Whitelock:‡ I was called to him on the evening of the same day: his house is situated in district 25, and is the last but one from Charles the Fifth's wall, and on the east side of flat Bastion road.

(Signed) "P. WILSON."

"To Dr. BARRY, Secretary to the Board."

Mr. Wilson in his Historical Sketch published in the Lancet in 1830, states that two individuals of a Jew family, residing on the floor over Whitelock's apartment, were taken ill anteriorly to him. He does not, however, quote any authority for this assertion. Mr. Leahy's four children are all returned officially on the 3d September, under the head of "bilious remittent," and all noted as belonging to the house in which Whitelock died.§ The Leahy and Whitelock families inhabited the same floor, and were particularly intimate.

(To be continued.) p. 96.

### OPIUM IN INFLAMMATION.

### *On the beneficial Effects of the External Application of Opium in Inflammatory Diseases.* By Dr. Bow.

In the work I lately published on the "Nature of Fever,"|| I endeavoured to draw attention to the external application of opium in that disease, as also in some inflammatory affections of the chest. I there stated my belief to be, that one of the

* Mr. Fraser, talking of the case of his own servant-maid, says, "I considered her disease at the time, (3d August,) in common with Mr. Peter Wilson, late of this hospital, to have been one of those sporadical attacks of *yellow fever* which every now and then appear here." (See Med.-Chirurg. Review, 1st September 1830, p. 345.)

† Mr. Wilson should have written *died*, instead of "lived;" for, when the woman was removed to the "low room," she had been already three days ill, and she died on the third day after her removal. (See Morning State, No. 1.)

‡ This man's Christian name was *John*. (See Morning State, No. 1.)—D. B.

§ See Morning State.

